# Field of Genes: An Investigation of Sports-Related Genetic Testing

**DOI:** 10.3390/jpm2030119

**Published:** 2012-09-12

**Authors:** Jennifer K. Wagner, Charmaine D. Royal

**Affiliations:** 1 Center for the Integration of Genetic Healthcare Technologies, University of Pennsylvania, 1112 Penn Tower, 399 S. 34th St., Philadelphia, PA 19104, USA; 2 Duke Institute for Genome Sciences & Policy, Duke University, 304 Research Drive, Box 90141, Durham, NC 27708, USA; E-Mail: charmaine.royal@duke.edu

**Keywords:** sports, athletics, genetic tests, ELSI, direct-to-consumer

## Abstract

Sports-related genetic testing is a sector of the diverse direct-to-consumer (DTC) industry that has not yet been examined thoroughly by academic scholars. A systematic search was used to identify companies in this sector and content analysis of online information was performed. More than a dozen companies were identified. Marketing practices observed generally did not target parents for child testing, and marketing images were mild compared to images used in popular media. Information was provided at a high reading level (industry-wide Flesh-Kincaid Grade Levels > 11). While ~75% of companies provide privacy policies and terms of service prior to purchase and ~40% provide scientific citations for their tests, <25% reported using American Association of Blood Banks (AABB) accredited or the Clinical Laboratory Improvement Amendments of 1988 (CLIA) certified laboratories. Tests ranged considerably in price (~$100–$1,100) and were substantively diverse. These findings highlight the need to appreciate nuances and avoid broad generalizations of this and other DTC sectors. Utilization of consumer protections available for e-commerce generally may adequately protect DTC genetics consumers without new federal legislation or regulation.

## 1. Introduction

The direct-to-consumer (DTC) genetic testing and personal genomic analysis industry is diverse, making the validity and utility of industry-wide generalizations and abstractions difficult to evaluate. As a result, scholarly attention has taken a sector-specific approach, analyzing companies that provide particular types of testing, such as nutrigenomic [1], ancestry [2], and health-related tests [3]. An area that has not received much specific scholarly attention (but see [4,5,6,7,8]) but has received increasing attention in popular media [9,10,11,12,13,14,15,16,17,18,19,20,21,22,23,24,25,26] is sports-related genetic testing. While the first sports-related genetic test appeared on the DTC market in 2004 [27,28,29] (just one year after a prominent research article reporting an association between elite athletic performance and ACTN3 genotype was published [30]), policy discussions and public debate continue to rely on speculation (often preoccupied with concerns surrounding testing of children [4,31,32,33] rather than on empirical data about the companies offering sports-related genetic testing, the specifics of the tests available, or the consumer transaction and experiences). 

To address this gap, we perform a systematic search to identify companies providing sports-related genetic tests and content analysis of these companies’ online practices and disclosures. We conclude with a discussion examining characteristics of the sports-related genetic testing sector as it relates to other DTC sectors, underscoring the importance of utilizing e-commerce protections already in existence to protect consumers. In the future, we will provide an examination of four sports-related tests through direct participant observation (highlighting test details, consumer transaction features, methods used to return results, and interpretations provided for consumers) [34].

## 2. Results

This investigation identified more than a dozen companies in the sports-related genetic testing sector, as shown in Table 1. Roughly half of the companies identified were based in the United States. Not all companies engaged in both DTC marketing and sales through their websites. CyGene Direct, for example, had been selling tests DTC and continued to market the product online at the time of our search, but hyperlinks provided on its website to purchase the product were no longer functioning, and reports indicated CyGene Direct had suspended its DTC sales [35]. As Table 2 highlights, the sports-related genetic tests available in May 2011 were considerably different from one another, reportedly involving analysis of between one and 60 genes. Prices ranged from below $100 for the “Monoamine Oxidase A (Warrior Gene)” product sold by Family Tree DNA to more than $1,100 for the “Sports DNA Test: Pro Genetic Test” sold by Advanced Healthcare Inc., India. Notably, higher prices were not indicative of an increased number of genes or loci included in the assay: AIBiotech’s “Sports X Factor Standard Panel” included a dozen genes for just $180 while the most expensive test offered included only one gene. For some companies, the sports-related genetic test was just one component of a broader personal genomic service (as was the case with 23andMe’s inclusion of the research report for “Muscle Performance” as one of the 50 traits reported, offered in addition to its many disease risk, carrier status, and drug response reports). For other companies, the sports-related genetic test was bundled with non-genetic products or services, such as training systems (e.g., “Atlas Pro” by Atlas Sports Genetics combined its genetic test with the EPIC Talent Identification System [36], a component of a strength and conditioning program that emerged from the University of Nebraska’s “Husker Power” program designed by Boyd Epley, Hall of Fame strength coach [37]). While all of the companies analyzed disclosed some information about the number and/or name of genes tested, information about the specific loci was difficult to locate, a problem documented in other sectors of the DTC industry (e.g., [1,2,3]). 

Marketing practices frequently included highlights of past media coverage of the companies’ products and services (e.g., occasions when the company was reviewed favorably in the press) and also included testimonials, endorsements, and feedback from previous consumers. A summary of other marketing practices—both prominent slogans and images—is provided in Table 3. Notably, our investigation uncovered inconsistent marketing practices for a company engaged in multiple sectors of the DTC industry. Family Tree DNA, one of the more prominent companies in the ancestry testing sector, suggested that its MAO-1 test would answer the question, “Are you a warrior?” [38]. While the company noted “[t]hese factoids are best used as ‘cocktail conversation’ starters” [39], the company’s use of Medieval armor imagery and its failure to provide a functioning hyperlink to the scientific citation it suggested is the foundation for its test (Note: The website stated, “Sabol *et al*., 1998 contains additional details about the ‘Warrior Gene’ variant,” but the hyperlink took the consumer to a page for general citations (at http://www.familytreedna.com/general-papers.aspx), among which there was no Sabol *et al*. 1998 paper, even when the user attempts to link to other “Available Categories” of paper.) may fuel critical suspicion of its sports-related test. Another unexpected result of this study—given the common criticisms surrounding sports-related genetic tests being offered for child testing—was that only three companies analyzed were found to engage in targeted advertising toward parents for testing of children. Moreover, a few companies provided recommended ages for their products, such as Athleticode (not intended for individuals under 13 years), Atlas Sports Genetics (not intended for individuals under 10 years), and My Gene (not intended for individuals under 18 years).

The results of website features that affect prospective consumers’ abilities to understand or navigate the online information are summarized as in Supplementary Table 1. Results were comparable to other DTC sectors [1,2,3]. Information content was provided at a relatively high reading level (with industry-wide mean Flesh-Kincaid Grade Levels above 11 for the home, test description, and purchase pages), regularly exceeding the 8th-grade reading level advocated as an upper limit for consumers and required by some consumer protection statutes [40]. Only one-third of the sites provided a glossary of terms, nearly two-thirds of companies provided a page for Frequently Asked Questions, and less than half provided search bars to facilitate navigation of the website information. No apparent customs have yet emerged from the sports-related sector for website organization, as the uses of and terms for header and footer navigational tabs are widely variable. 

**Table 1:** Companies providing sports-related DNA tests or analyses.

Company	Website	Approx. Year Company Began	Location	Related Companies
23andMe, Inc.	www.23andme.com	2006	CA, USA	
Advanced Health Care Inc., India	http://www.advanceddna.in/sports.aspx	2008	India	
American International Biotechnology Services (AIBiotech)	http://www.sportsxfactor.com/Home.aspx	2010	VA, USA	Botswick Laboratories, Inc.
Asper Bio Tech	http://www.asperbio.com/athletic-gene-test	1999	Estonia	Estonian Biocentre
Athleticode, Inc.	www.athleticode.com	2009	CA, USA	
Atlas Sports Genetics, LLC	http://www.atlasgene.com/	2008	CO, USA	Zybek Sports, LLC; Zybek Athletic Products, LLC; Genetic Technologies Ltd; Epic Athletic Performance
Cosmetics DNA	http://www.cosmetics-dna.com/questions_answers.htm#I_want_to_test_my_DNA	unknown	Israel	UmaPuri Ltd; Dr. M. Burstein Ltd.; Bio Anti Aging Ltd.; Dr. Burstein Dead Sea Ltd., (DBS)
CyGene Direct *	http://www.cygenedirect.com/browse-10873/Optimum-Athletic-Performance-Dna-Analysis.html	2003	FL, USA	CyGene Laboratories Inc.
DNA4U *	http://www.gonidio.com/test.php?id=2	unknown	Greece	Gonidio
Family Tree DNA	http://www.familytreedna.com/Default.aspx	2000	TX, USA	Genealogy by Genetics, Ltd.
Genetic Technologies Limited *	http://www.gtpersonal.com.au/sports_performance.php	1989	Australia	
My Gene *	http://www.mygene.com.au/product/sport-genetic-test	unknown	Australia	
Warrior Roots	www.warriorroots.com	2008	MD, USA	Sorenson Genomics

List was prepared May 2011. * Indicates companies that, while clearly marketing direct-to-consumer (DTC), may not be selling DTC, as suggested by broken or nonfunctioning hyperlinks on company websites or absence of online purchase options.

**Table 2:** Summary of sports-related DNA products.

Company	Product Name	Price	Number of Markers Tested	Markers Tested
**23andMe, Inc.**	“23andMe Kit” (“Muscle Performance” is a trait included as a 4-star “established research report”)	$399.00	1	ACTN3
**Advanced Health Care Inc., India**	“Sports DNA Test: First Genetic Test”	$275.09	1	ACTN3
	“Sports DNA Test: Pro Genetic Test”	$1,127.37	1	ACTN3
**American International Biotechnology Services (AIBiotech; SportsXFactor)**	“Sports X Factor Standard Panel”	$180.00	12	ACTN3, ACE, PPARGC, DI01, VEGFR, NOS3, IL6, APoE, HCM (MYH7, MYBPC3, and TNNT2), SCN5A
	Sports X Factor Standard Panel with all add-on options (“additional ACL/Soft Tissue Injury Panel”; “additional Hereditary Hemochromatosis”; “additional Cardiac Marker Panel”; and “additional Customized Workout”)	$900.00	17	ACTN3, ACE, PPARGC, DI01, VEGFR, NOS3, IL6, APoE, HCM (MYH7, MYBPC3, and TNNT2), SCN5A; COL1A1, COL5A1, COL12A1, TNC, and MMP3
**Asper Bio Tech**	“Athletic gene test”	$118.64	2	ACE and ACTN3
**Athleticode, Inc.**	“Race Time Kit”	$79.99	1	COL5A1
	“Body Scope Kit”	$189.99	5	COL5A1, COL1A1, COL12A1, MMP3 and GDF5
**Atlas Sports Genetics, LLC**	“Atlas First”	$169.99	1	ACTN3
	“Atlas Pro”	$999.99	1	ACTN3
**Cosmetics DNA**	“Athletic Performance”	$519.75	“60 Genes/ 79 SNPs”	n/d
**CyGene Direct**	“Optimum Athletic Performance DNA Analysis”	$99.95	5	ACE, APoE, B2R+9/-9, eNOS, VDR BSM1, VDR FOK1
**DNA4U**	Athletic Performance	$419.00	“60 Genes/ 79 SNPs”	n/d
**Family Tree DNA**	“Factoid Tests: Muscle Performance”	n/d	1	ACTN3 (disclosed as “rs1815739 SNP”)
	Monoamine Oxidase A (Warrior Gene)	$99.00	1	MAO-1
**Genetic Technologies Limited**	“ACTN3 Sports Gene Test”	$214.93	1	ACTN3
**My Gene**	“Sports Gene Test”	n/d	18	n/d
**Warrior Roots**	“Athletic Profile Test”	$199.95	9	ACTN3, MCT1, HIF1, ADRB2, DIO1-D1a, DIO1-D1b, NOS3, PPARGC1A, ACE

n/d = not disclosed. Price provided is in U.S. Dollars and reflects the price as advertised on May 2, 2011.

**Table 3:** Marketing of sports-related DNA products.

Company	Prominent Marketing Slogan	Prominent Marketing Images
23andMe, Inc.	“Start filling in the gaps with your DNA”	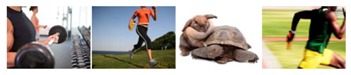
Advanced Health Care Inc., India	“Bringing you the world of genetics”	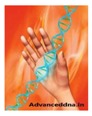
American International Biotechnology Services (AIBiotech; SportsXFactor)	“Test Enables Individuals to Customize Workout Programs Based on Genetic Results”; “New Genetic Test Helps Athletes Maximize Performance, Identify Undiagnosed Risk Factors”	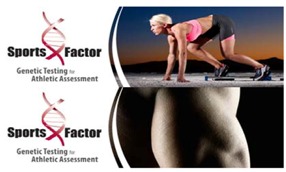
Asper Bio Tech	“Athletic performance test of strength, speed and endurance”	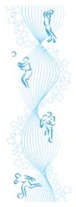
Athleticode, Inc.	“DNA Testing to Prevent Injuries & Improve Performance” and “Get Tested. Get Your Results. Get Going!” and “The world of athletics is undergoing a sea of change. And Athleticode is leading the way”	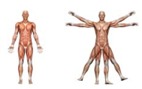
Atlas Sports Genetics, LLC	“Let us help you become the best athlete you were born to be”	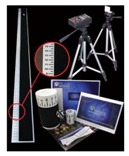
Cosmetics DNA	“Achieve optimal sport performance”	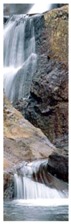
CyGene Direct	“Our athletic & sports analysis will help you learn more about your athletic gene as well as help you improve athletic performance” and “CyGene's Optimum Athletic Performance DNA Analysis can help you assess what type of sport or event you are genetically wired for and what sports put you at increased risk of physical or neurological injury.”	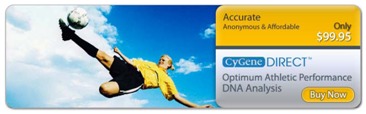
DNA4U	“Identify your athletic strengths and weaknesses”	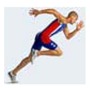
Family Tree DNA	“Are you a warrior? IN SPORTS OR BUSINESS, HOW DO YOU RESPOND TO STRESS? IS THE ANSWER IN YOUR GENES?”	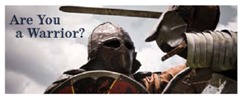
Genetic Technologies Limited	“This test provides an insight into the way an individual is genetically predisposed, be it for sprint-power or endurance type sports using DNA technology.”	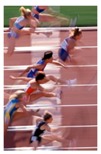
My Gene	“For optimum training and performance results”; “Whether you are just starting out with a new exercise program, you enjoy playing sport for a local club, or if you are an elite athlete, taking a Sport and exercise genetic test will open the door to more personalised, effective exercise training and performance.”	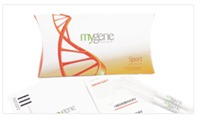
Warrior Roots	“The Athletic Profile Test outlines your natural genetic performance strengths, and gives insights into how to maximize your potential.”	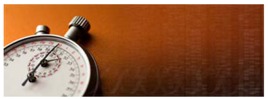

All images retrieved from product description or purchase pages on May 2, 2011.

Table 4 summarizes the scientific and legal disclosure customs. Notably, less than one-quarter of the companies reported using American Association of Blood Banks (AABB) accredited or the Clinical Laboratory Improvement Amendments of 1988 (CLIA) certified laboratories, and less than one-quarter provided prospective consumers with a sample results report prior to purchase. Approximately 40% of the companies directed consumers to the scientific journal articles used as the foundation for their tests. Roughly three-quarters of the companies provided their privacy policies and terms of service to prospective consumers for review prior to purchase, and these policies were found under easily recognizable headings. Provisions for forum selection (*i.e*., a provision setting the location where any disputes would be adjudicated, such as any state or federal court located in County Marin, CA as set by Athleticode) and choice of law (*i.e*., a provision establishing which jurisdiction’s laws would be applicable to adjudicate any disputes such as laws of the State of Colorado, as set by Atlas Sports Genetics) were commonly but not universally included in the terms of service. Explicit disclaimers of the implied warranties of merchantability and fitness for particular use (two advantageous terms for consumers) were included in only half of the terms of service posted online. Interestingly, only three companies (23andMe, AIBiotech, and Athleticode) made it clear to prospective consumers that informed consent is required as part of the purchase process.

**Table 4:** Scientific and legal disclosures.

Item of Interest	Number of Companies (Proportion of Industry, N = 13) Disclosing Item
***Scientific Disclosure Items***	
AABB Accreditation	3 (23.1%)
CLIA Accreditation	3 (23.1%)
Sample Results Report	3 (23.1%)
Journal Article References That Form Scientific Basis of the Products	5 (38.5%)
Members of Scientific or Corporate Advisory Boards	10 (76.9%)
***Legal Disclosure Items***	
Privacy Policy	10 (76.9%)
Terms of Service	10 (76.9%)
Forum Selection Provision	5 (38.5%)
Choice of Law Provision	6 (46.2%)
Specific Disclaimer of the Implied Warranty of Merchantability	5 (38.5%)
Specific Disclaimer of the Implied Warranty of Fitness for Particular Use	5 (38.5%)
Cap on Potential Damages	7 (53.8%)

It is important to note that all disclosure items listed here are available for the prospective consumer’s review prior to the purchase of a product. Additionally, instances of positive disclosure are shown here, not negative disclosure. For example, the number of companies disclosing AABB accreditation indicates the number of companies with websites expressly stating that the lab has AABB accreditation (and does not include companies with websites expressly stating that the lab lacks AABB accreditation, if such negative disclosures exist).

Table 5 summarizes the prominent benefits, risks, and limitations of sports-related genetic testing as provided by the companies to prospective consumers prior to purchase. While 38.5% of the companies discussed “benefits” explicitly, only 15.4% discussed “risks” explicitly. Only three companies stated that a benefit of testing is that it would help determine which sports the consumer should play. Limitations were discussed by more than two-thirds of the companies, with companies often using identical, boilerplate language (e.g., Cosmetics DNA, DNA4U, and Warrior Roots all stated, “[t]he information provided by [company] is neither comprehensive nor absolute, and may not be applicable to individual circumstances should the information be subsequently deemed inaccurate or out of date”). In addition to general statements that the test is provided “as is,” some companies (e.g., AIBiotech) warned consumers that genetic factors are “only one part of the picture” and information reported does not speak to the genetic markers and other risk factors not tested. Compared to other companies, Athleticode provided the clearest statement of the current limitations to sports-related genetic testing in Item 2.4 of its Terms and Conditions, which stated,

“The risk information provided by the Athleticode Player Report and Code Nation services are based on current scientific knowledge, and this information is changing and improving over time. Your estimated risk for specific conditions is based on what is currently known about genetic contributions to these conditions. Over time, new studies are likely to be published that would change your risk estimates. Currently, most of the published studies in this area of genetic research have focused on people of Western European descent. We do not know if, or to what extent, these results apply to people of other backgrounds. Our risk estimates do not account for the impact of your behavior, lifestyle, and environment on your chance of developing injuries or athletic conditions. However, genetic, behavioral, lifestyle and environmental factors (such as your playing environment) all contribute to your overall risk of injury or developing a particular condition. Our statements regarding possible injuries account only for genetic influences.”

**Table 5:** Stated benefits, risks, and limitations of sports-related genetic tests.

Benefits	# (%)	Risks	# (%)	Limitations	# (%)
Gives you insight; Gives you information; Reveals your genetic performance indicators and risk factors; Knowledge is empowering	9 (81.8%)	You may learn unanticipated things about yourself	2 (15.4%)	Not medical advice; not intended for medical purposes or clinical diagnosis or patient mgmt decisions	7 (53.8%)
Helps you adjust your training regimen to reduce risk of certain injuries and improve performance	7 (53.8%)	Learning new or surprising information may cause you “unforeseen emotional responses”	1 (7.7%)	Results are not absolute; Genetic factors are only a component; environmental factors are important	7 (53.8%)
Makes it easier to choose the most suitable sport for you	3 (23%)	“Knowledge is irrevocable.”Once you know, you know. There is no un-ringing the bell.	1 (7.7%)	Test is not comprehensive, does not rule out all possibilities	5 (38.5%)
Helps you maximize/optimize/enhance performance	5 (38.5%)	Test results/information you share with others might be used against your interests	1 (7.7%)	The information provided is based on current scientific understanding; the information is changing; the information may become inaccurate or outdated	5 (38.5%)
Understand nutritional requirements specific to you	1 (7.7%)	There may be problems during sample processing that may result in errors in your test results	1 (7.7%)	Results are based on studies that mainly focused on people of European ancestry; may not be applicable to individuals of non-European ancestry	2 (15.4%)

# indicates the number of companies that discussed this stated benefit, risk, or limitation. (%) indicates the proportion of companies (N = 13) that stated this benefit, risk, or limitation.

## 3. Methods

To identify companies marketing and selling sports-related genetic tests, a Google search was performed on May 2, 2011 using the keywords arranged into the following 12 search strings: DNA + athletic; DNA test + sports; DNA test + athlete; DNA test + muscle performance; genetic test + sports; genetic test + athlete; genetic test + muscle performance; genetic analysis + athletic; genetic analysis + athlete; genetic analysis + sport; “DNA testing in athletes”; and “DNA testing for athletes.” For each search, the first 25 search results retrieved were reviewed to identify companies that appear to sell sports-related genetic tests. Thus, a total of 300 search results were reviewed. While this approach systematically identified companies, it may not have identified all possible companies. [Note: Upon reviewing literature one month after the initial search and data collection (on June 3, 2011), an additional company, My Gene Profile, was discovered that was not identified in the initial search and, thus, was not included in the content analysis. This company is distinct from My Gene, a company that was identified and included in the analysis. Because this investigation is a snapshot of this dynamic industry using the stated methods (methods which did not identify this company), the authors feel that to collect data and include My Gene Profile in the content analysis *post-hoc* would introduce unascertainable error, since the authors would have no way of knowing what (if any) of the data available on the company’s website on or after June 3, 2011 were available on May 2 or 3, 2011, the original dates used for data collection of all other companies.] For example, Google returns search results by “relevance” as informed by earlier search habits of the user (whether signed-in or out), which may introduce coverage or selection bias in the identification of companies. To minimize this potential for systematic bias and to maximize the number of companies identified, when the search result was a uniform record locator (URL) that did not belong to a testing company’s direct domain (but was, for example, a news article, press release, or blog post), the URL was reviewed to identify companies mentioned within that search result. Websites for companies identified were archived with Internet Researcher 2.1, developed by Zylox Software, Inc. 

Data were collected from each of the companies’ websites on May 2–3, 2011 by one researcher, and the same researcher coded each company’s website coded independently for content analysis, including the following variables of interest: organization characteristics (company name; date upon and state in which articles of organization, incorporation or fictitious name of business were filed; location; names of any related or companion companies; registered agent and address); product characteristics (product name; price as advertised; price converted to US Dollars; number of loci and genes included in the analysis; and bundling of non-DNA products or services with the DNA analysis); marketing characteristics (prominent marketing slogans and images; directed marketing to parents, pre-college athletes, college athletes, professional athletes, coaches, or trainers; notable quotations); literacy and navigational characteristics (readability of the home page, test description page, and purchase page; presence of a glossary, search bar, FAQ page, and video tutorials; and the names of header and footer tab titles); scientific disclosures (accreditation by the AABB, certification pursuant to CLIA, or other accreditations and certifications); presence of a sample report prior to purchase; scientific citations for the test; identification of members of scientific consultants or a corporate board); legal disclosures (availability of terms of service and privacy policies prior to purchase; title of terms of service and privacy documents; main topics covered in privacy policies; practices regarding “informed consent”; forum/venue selection; choice of law provisions; implied warranties of merchantability and fitness for a particular use; caps on damages; language used to disclaim warranties and otherwise limit liability); and the acknowledged limitations, risks, and benefits of the sports-related genetic tests offered. To assess readability, content was selected and copied from the specific page of interest and pasted into a Microsoft Word document, where the review function with readability statistics was utilized. 

## 4. Conclusions

With no “typical” sports-related test being offered and the absence of clearly established industry customs, the data reported here underscore the need for a nuanced appreciation of the sports-related genetic testing sector. More than a dozen companies were identified, and a vast array of tests was observed (with tests comprising analysis of a single gene to reportedly 60 genes). Thus, it was apparent that the DTC sports-related genetic testing sector was offering more than just *ACTN3* results and predictions of whether a consumer is a better power or endurance athlete, though that topic seems to receive the bulk of the attention from the popular press. Marketing strategies were not found to be directed at parents to identify the next superstar athlete or determine how children should be raised. Advertising slogans and images of the companies investigated were rather mild when compared to the sensationalized headlines and startling images of the popular media’s coverage (e.g., the news article titled “Baby Olympian? DNA test screens sports ability” accompanied by a picture of a baby face situated atop a double helix transforming into muscle-flexing arms) [31]. As reported here, the majority of companies investigated indicated these tests are not for medical purposes or decisions and results are not absolute. Many companies reminded consumers that the underlying science is continually changing and subject to revision, and some reminded consumers that genetic factors are just one part of the explanation for variation in athletic abilities and performance. Criticisms of these DTC tests have included that the products are “snake oil” and are fraught with genetic deterministic messages [32]. The results of this investigation, however, suggest these criticisms are overstated—which is not to say that the sector is undeserving of any criticism or that the sector has no room for improvement of its practices. Additional research is warranted to understand (a) how well prospective and actual customers grasp the caveats made by the companies; (b) what the customers’ expectations, motivations, intended uses for such tests are; and (c) customer reactions and satisfaction to their purchases of sports-related DNA tests. Such data are vital to development of appropriate and effective policies. 

The Food and Drug Administration (FDA) has shown increased interest in oversight of DTC genetic tests since 2010, and experts have engaged in considerable debate regarding how the FDA could feasibly and effectively oversee DTC genetic tests [41,42,43,44,45,46,47,48,49,50,51,52,53,54,55,56]. No consensus has emerged, leaving the DTC industry, including its sports-related genetic testing sector, in regulatory limbo. In June 2011, the FDA issued draft guidance and sought comments on how it might oversee products labeled for research use only (RUO) or investigational use only (IUO) [57], labeling commonly used in the DTC marketplace. The FDA has not yet issued clear guidance on how they will oversee the various sectors of the diverse DTC industry—particularly those sectors with companies whose terms of purchase explicitly indicate the tests or services are not intended for medical use. For a variety of legal and policy reasons, it is unlikely that the FDA will exercise oversight of the ancestry sector. However, in light of the FDA’s letter to AIBiotech requesting justification for its Sports X Factor test [58], it seems apparent that the FDA will treat the sports-related sector less like the ancestry sector and more like the health-related sector. 

The American Bar Association passed a resolution in 2011 that urges federal, state, and territorial governments to take action against DTC companies offering health-related tests (termed “direct-to-consumer medical genetic testing” or “predictive and diagnostic medical genetic testing” in the resolution) if the companies do not meet a number of requirements, including processing of samples in a CLIA certified laboratory, having results and interpretations reviewed and authorized by a “qualified health personnel” prior to release to the consumer, making full disclosure of material facts related to the tests, advising consumers of risk of disclosure of personal information, taking measures to protect security and confidentiality of the information, and advertising truthfully and accurately [59]. It is yet to be seen how local, state, and federal officials will respond to this resolution.

The sports-related genetic testing sector and DTC industry as a whole are not unconstrained, even in the absence of a specific federal law or regulation that mandates particular standards for DTC genetic tests. It is both impractical and unnecessary to create a new legal framework every time a new test or service enters the market. The DTC industry is constrained by laws and customs applicable to commerce generally. Consumers who have had a bad experience or are dissatisfied with a company’s practices have at their disposal the right to take legal action and seek recovery for damages on a variety of contract and tort principles. There are statutory protections afforded under both federal consumer protection statutes (e.g., the Federal Trade Commission, or FTC, Act) and state-specific consumer protection statutes (sometimes called “little-FTC acts,” which may offer broader, more distinct consumer protections than federal statutes [60]). These enable federal and state governmental officials to track complaint trends, weigh the enforcement priorities as driven by the data (e.g., the number and urgency of complaints regarding DTC genetic testing may be minor compared to complaints in other areas of commerce) rather than speculation, and appropriately distribute resources to enforce existing laws for the maximum benefit of all consumers. In our view, only if those existing remedies prove themselves to be inadequate should new legal remedies be recommended. 

It seems apparent that laws and regulations as applied to e-commerce practices are under-enforced [61]. This is not a problem unique to DTC genetic testing and, accordingly, the problem requires solutions also not unique to genetic testing. Like other areas of e-commerce aptly described by legal scholar Peter Swire [61,62,63], the personal genomics industry (and its sports-related sector) will consist of both elephants (businesses that are too big to ignore, have thick skins, and are able both to avoid liability and lobby effectively) and mice (numerous small businesses able to hide, nest, multiply rapidly, and evade enforcers). As a result, a multifaceted (not necessarily federal) approach to protect consumer interests may be preferable.

The data reported here are valuable resources that should both encourage and enable future research on a variety of issues surrounding sports-related genetic testing (e.g., what consumers purchasing sports-related genetic tests hope to achieve; how these tests are currently or might eventually be applied in personalized genomic training regimens for amateur and professional athletes; how personal genomics might be applied to improve athlete safety; and how personal genomics implicates and challenges sport doping and competitive fairness policies).

## Supplementary Files

Supplementary File 1 PDF-Document (PDF, 99 KB)
