# Supplementary material for: Field of Genes: An Investigation of Sports-Related Genetic Testing
Source: J Pers Med. 2012 Sep 12;2(3):119–37. doi: 10.3390/jpm2030119 (PMC4251368; doi:10.3390/jpm2030119)
Supplement: Supplementary File 1 — PDF-Document (PDF, 99 KB) [file jpm-02-00119-s001.pdf]

Supplementary Table 1. Website features relevant to consumer understanding of sports-related DNA products.

Company	Website Readability (Flesh-Kincaid Grade Level)			Literacy Tools			Website Navigation Tools		
	Home Page	Purchase Page	Product Page	Glossary	Video Tutorials	FAQ Page	Search Bar	Header Tab Titles	Footer Tab Titles
23andMe, Inc.	8.9	10.5	10.5	Yes	Yes*	Yes	Yes	welcome; ancestry; health; how it works; store (also login; register your kit; blog; help; cart)	Blog; About us; Jobs; 23andWe; Terms of Service; Privacy Statement; Consent Document
Advanced Health Care Inc., India	12.9	12.1	12.1			Yes		Home; About us; DNA Testing Services; Fee Schedule/Payment; Sample Collection; Bank Details; FAQ; Request Info; Site Map; Contact Us; E-Client	None
American International Biotechnology Services (AIBiotech; SportsXFactor)	11.9	9.4	12.4					Home; Test Information; Order; About Us; Contact Us	Privacy Policy; Legal Notice
Asper Bio Tech	17.1	12.2	13.8				Yes	About AsperBiotech; Genetic Tests; Ordering; Science; Fp7 Projects; Media Center; Partnership; Genetics; Athletic Gene Test	None
Athleticode, Inc.	11	8.8	10.3					Home; How It Works; What we Offer; Get Started (also About Us; Contact Us; Register Your Kit; Log in)	None
Atlas Sports Genetics, LLC	10.8	12.4	6.1				Yes	Home; Products; Information; Contact Us; My Account; Shopping Cart	Home; Privacy Policy; Conditions of Use; Disclaimer; Links
Cosmetics DNA	14	9.6	9.6	Yes		Yes		Home; Products; Q&A; Our Quality; Your Privacy; Career; Contact	Home; Products; DNA Test Sample; Your Derma Genes; Q&A; About Us; About our Quality; Catalogue; Glossary; Consult Us; Career; Contacts; Links

Supplementary Table 1. *cont.*

**CyGene Direct**	11.8	13.3	13.3			Yes		Home; Blog; RSS; Login; About CyGene; Contact; Espanol; Russian; Resource Center; Genetic Testing; For Professionals; Shop	Home; Blog; RSS; Login; About CyGene; Contact; Espanol; Russian
**DNA4U**	8.7	12.7	12.7	Yes		Yes		Home; DNA Tests; About Us; Knowledge Base; FAQ; Contact	Terms of Use; Privacy Policy; Security; Contact Us
**Family Tree DNA**	10.9	12.8	9.3	Yes	Yes*	Yes	Yes	Home; Products; Projects; Testimonials; FAQ; About; Feedback	Home; Products; Projects; Testimonials; FAQ; About; Login; Careers; Privacy; Sitemap; Contact Us
**Genetic Technologies Limited**	13.1	10.9	10.9				Yes	medical, forensics, paternity, personal, animal	links, contact us, disclaimer, terms and conditions, privacy, sitemap
**My Gene**	10.1	11.7	13			Yes	Yes	Home; About Us; Genetic Tests; Products; Information; Contact Us; Terms (Also View Your Results; Medical Section; and FAQs)	About us; FAQs; Ordering; Contact us; Sport; Weight Loss; Coeliac; Statin; Lactose; MyGene Medical; Terms & Conditions; Your Privacy; Returns & Refunds; Delivery
**Warrior Roots**	8.9	10.3	9.4			Yes		Home; Warrior; Athlete; Store; About; Contact	None
**(Number of Companies) Proportion of Industry Providing Feature**	**(3**)** **23.1%**	**(1**)** **7.7%**	**(1**)** **7.7%**	**(4)** **30.8%**	**(2*)** **15.4%**	**(8)** **61.5%**	**(6)** **46.2%**	**(13)** **100%**	**(9)** **69.2%**

* Video Tutorials are offered by company but not video tutorials specific to the sports-related DNA product.

**A company was considered to meet readability requirements if it provided the information at an 8th Grade Reading Level, which included any scores not exceeding 8.99.
